# Estimation of positron emission tomography amyloid load and related biomarkers in Alzheimer’s disease using evoked potential tomography EEG: development and internal validation in a cross-sectional cohort

**DOI:** 10.1186/s13195-026-02076-7

**Published:** 2026-06-12

**Authors:** Boris-Stephan Rauchmann, James Hamet, Jesyin Lai, Homeira Kafi, Joe Rexwinkle, Matthias Brendel, Nicolai Franzmeier, Carolin Kurz, Oliver Pogarell, Johannes Levin, Günter Höglinger, Robert Perneczky

**Affiliations:** 1 Department of Psychiatry and Psychotherapy, LMU Hospital, Ludwig-Maximilians- Universität Munich, Munich, Bavaria 81377 Germany; 2 German Center for Neurodegenerative Diseases (DZNE) Munich, Munich, Bavaria 81377 Germany; 3 Sheffield Institute for Translational Neuroscience (SITraN), University of Sheffield, Sheffield, South Yorkshire S10 2HQ UK; 4 NeuroImaging Core Unit Munich (NICUM), University Hospital LMU, Munich, Bavaria 80336 Germany; 5 Vistim Labs, Inc, Claymont, Delaware 19703 USA; 6 Department of Nuclear Medicine, LMU Hospital, Ludwig-Maximilians-Universität Munich, Munich, Bavaria 80336 Germany; 7 Munich Cluster for Systems Neurology (SyNergy), Munich, Bavaria 81377 Germany; 8 Institute for Stroke and Dementia Research, LMU Hospital, Ludwig-Maximilians- Universität Munich, Munich, Bavaria 80336 Germany; 9 Department of Psychiatry and Neurochemistry, The Sahlgrenska Academy, Institute of Neuroscience and Physiology, University of Gothenburg, Gothenburg, 41345 Sweden; 10 Department of Psychiatry and Psychotherapy, Division of Mental Health of Older Adults, LMU Hospital, Ludwig-Maximilians-Universität Munich, Nußbaumstr. 7, Munich, Bavaria 80336 Germany; 11 Department of Neurology, LMU Hospital, Ludwig-Maximilians-Universität Munich, Munic, Bavaria 80336 Germany; 12 Ageing Epidemiology (AGE) Research Unit, School of Public Health, Imperial College London, London, W2 1PG UK

**Keywords:** Alzheimer’s disease, Electroencephalography, Event-related potential, PET-amyloid SUVR, AD biomarkers, Regression modeling

## Abstract

**Background:**

Dementia affects over 50 million individuals globally, predominantly due to Alzheimer’s disease (AD). Effective early detection and intervention remain clinical challenges, as there is a lack of unified, portable solutions to assess multiple biomarkers.

**Methods:**

We evaluated Evoked Potential Tomography (EPT), an EEG-based method using a novel visual evoked potential protocol. An automated pipeline for EEG preprocessing, ERP extraction, feature selection, optimization, and regression modeling was developed to estimate key AD biomarkers: PET-amyloid standardized uptake value ratio (SUVR), CSF phosphorylated tau (*p*-tau181), Free and Cued Selective Reminding Test (FCSRT), and Mini-Mental State Examination (MMSE) scores.

**Results:**

Regression models using ERP features from dementia participants demonstrated strong correlations (*r* = 0.8–0.94, *p* < 0.01) between predicted and true PET-amyloid SUVR, *p*-tau181, FCSRT, and MMSE values. In an independent external cohort, PET-amyloid SUVR predictions remained significantly associated with true values (*r* = 0.60, *p* < 0.01).

**Discussion:**

Despite limitations, these preliminary results support EPT’s potential as a sensitive and non-invasive method for estimating AD-related biomarkers in a clinically enriched AD cohort. Further validation studies are ongoing.

**Supplementary Information:**

The online version contains supplementary material available at 10.1186/s13195-026-02076-7.

## Background

Analyses of brain tissue from human and animal models suggest that fibrillar amyloid-β (Aβ) negatively impacts both excitatory neurons and inhibitory terminals, disrupting neuronal function and causing network-level damage [1, 2]. Research involving transgenic mice with Alzheimer’s Disease (AD) overexpressing Aβ demonstrates that neuronal hyperactivity in cortical and hippocampal regions, disruption of slow-wave oscillations, and network hypersynchrony precede plaque formation, indicating that hyperactivity may be one of the earliest physiological disturbances in AD pathogenesis [3, 4]. Additionally, phosphorylated tau (*p*-tau) impairs microtubule structure in axons both in vitro and in mice [5], resulting in a progressive reduction in synaptic quantity and efficiency, and disrupting intra- and inter-regional brain communication [6]. Moreover, recent findings suggest that cerebrospinal fluid (CSF) *p*-tau significantly modulates neuronal excitability and network activity in AD and related tauopathies [7].

Alterations in neural activity observed in animal models can now be examined in humans through electroencephalography (EEG), particularly through resting-state functional brain network analysis and event-related potential (ERP) analyses. These methods reveal changes in functional connectivity even in healthy aging individuals [8] and prodromal AD cases [9]. Despite advancements in connectivity analysis, EEG remains underutilized in standard AD diagnostics. Given the cost-effectiveness, non-invasiveness, and widespread availability of EEG equipment in clinical settings, EEG analysis represents a promising tool for population-wide AD screening and early risk detection. Previous studies have shown correlations between CSF Aβ status and EEG connectivity measures in cognitively normal older adults [10]. Additionally, patients with mild cognitive impairment (MCI) who are CSF Aβ-positive exhibit increased global slowing in brain oscillatory activity compared to Aβ-negative controls [11]. Evidence also links tau pathology to network disruptions detected by magnetic resonance imaging (MRI) in fully symptomatic AD [12] and other neurodegenerative diseases like progressive supranuclear palsy [13]. Recent developments in machine learning and artificial intelligence have facilitated automated assessments of EEG data, enabling methods that simultaneously learn meaningful features from the data and classify disease states [10].

Furthermore, recent research has demonstrated that EEG analysis can achieve high accuracy in distinguishing individuals at risk for AD from healthy controls [14]. Novel approaches include connectivity measures such as coherence or phase lag and graph analysis characteristics combined with data-driven machine learning and deep learning models, which enhance the detection and classification of neurological and mental health disorders [10, 15, 16]. Independent studies report that individuals with subjective cognitive decline, the earliest clinical stage of AD, who are positive for Aβ, exhibit enhanced functional resting-state connectivity in the alpha frequency band correlated with higher global Aβ load and reduced connectivity in the beta frequency band [17, 18]. However, the direct association between early electrophysiological changes and pathological hallmarks of AD remains to be firmly established through rigorous statistical and machine learning methodologies.

In addition to pathological biomarkers such as amyloid and *p*-tau, clinical cognitive assessments are essential tools in evaluating memory and overall cognitive functioning in AD research. Among these assessments, the Free and Cued Selective Reminding Test (FCSRT) and the Mini-Mental State Examination (MMSE) are widely utilized due to their sensitivity and practicality. The FCSRT specifically assesses verbal episodic memory and has proven particularly useful in characterizing memory impairments within the AD spectrum [19]. For instance, patients exhibiting lower FCSRT free recall scores demonstrate higher prevalence rates of dementia and an increased risk of progressing to dementia in the future [20]. Complementing the FCSRT, the MMSE is frequently employed as a screening tool for dementia, predicting cognitive decline [21], and monitoring cognitive changes over time [22].

Thus, the primary aim of this study was to investigate the associations between visually evoked responses captured by EEG and key pathological changes and clinical cognitive scores in individuals with AD, MCI, and age-matched controls within a prospective clinical study. We utilized a novel Evoked Potential Tomography (EPT) protocol comprising optimized visual stimuli that systematically varied in contrast, spatial frequency, and relative movement to selectively activate distinct neuronal populations involved in early visual processing. A similar protocol has previously been shown to effectively differentiate between patients with MCI and AD, revealing distinct spectral features correlated with continuous PET-amyloid SUVR [23]. Moreover, this protocol has demonstrated sensitivity to cognitive variations in schizophrenia and autism spectrum disorders [24, 25], highlighting its potential as a broadly applicable tool for assessing neural processing integrity.

Visual stimulation paradigms that probe early sensory processing have a long history of use in neuropsychiatric and neurodegenerative research, as visual ERPs are sensitive to synaptic dysfunction [26], inhibitory–excitatory imbalance [27], and altered cortical gain control [28]. Paradigms manipulating contrast, spatial frequency, and motion have been shown to reveal early dysfunction in disorders [29–31] including AD, schizophrenia, autism spectrum disorders, and Parkinson’s disease, often preceding overt cognitive decline. The EPT protocol builds upon this established literature by systematically engaging distinct neuronal populations within early visual pathways, thereby enhancing sensitivity to subtle network-level dysfunction associated with early pathological changes. Consequently, we hypothesized that EEG features extracted during EPT could accurately estimate PET-amyloid SUVR, *CSF p*-tau181 values, and other cognitive scores, such as FCSRT free recall and MMSE, across individuals at varying stages of dementia.

## Methods

### Study design and participants

The data used in this study originate from the baseline dataset of the ActiGliA study, a prospective, longitudinal observational study within the Munich Cluster for Systems Neurology (SyNergy) at Ludwig-Maximilians-University (LMU) Munich, initiated in 2017 [32]. Participants analyzed in this study were recruited through a specialized outpatient clinic at the LMU hospital Department of Psychiatry and Psychotherapy. Participants were included after providing written informed consent according to the Declaration of Helsinki. The study was approved by the ethics committee of LMU Munich (project numbers 17–755 and 17–569). ActiGliA includes extensive assessments that cover neurocognitive evaluations, clinical examinations, MRI and PET imaging, as well as bio-banking of fluids, including CSF testing. Further details on the study design, participant characteristics, and methods are described elsewhere [32]. Out of 140 ActiGliA participants, 48 of these individuals had EEG recordings available. This resulted in a cohort consisting of 16 participants with AD, 8 participants with MCI (cognitive decline not meeting criteria for dementia), 22 age-matched control participants with close-to-normal neurodegeneration CSF biomarkers (amyloid and *p*-tau), and 2 participants with non-Alzheimer’s dementia (NAD). Demographic information for these participants is summarized in Table 1.

**Table 1:** Demographic info of participants with EEG recordings

Measure	AD	MCI	Control	NAD	All
Sex *n*	F: 11; M: 5	F: 3; M: 5	F: 11; M: 11	F: 2; M: 0	F: 27; M: 21
Age					
* n*	16	8	22	2	48
* median*	73	67.5	71.5	71	72
0.5*IQR	3.38	7	2.88	2.5	5
PET-amyloid SUVR					
* n*	16	8	22	2	48
* median*	1.62	1.63	1.04	1	1.42
0.5*IQR	0.08	0.12	0.06	0.02	0.3
CSF *p*-tau181					
* n*	13	8	19	2	42
* median*	97.75	88.18	52.36	47.66	69.59
0.5*IQR	10.17	28.45	14.16	6.01	21.56
CERAD score					
* n*	16	8	22	2	48
* median*	52.25	72.5	86.5	51.75	73
0.5*IQR	5.56	5.31	3.25	2.88	14.94
FCSRT free recall score					
* n*	12	4	21	1	38
* median*	7	7.5	28	6	21
0.5*IQR	5.88	3.12	2.5	0	9.38
MMSE score					
* n*	16	8	22	2	48
* median*	23	28	29	23	28
0.5*IQR	1.5	1.25	0.5	1	2.62
CSF amyloid ratio					
* n*	12	8	19	2	41
* median*	0.04	0.04	0.07	0.1	0.05
0.5*IQR	0.01	0.01	0.01	0.01	0.02

*AD*  Alzheimer’s disease, *NAD*  Non-Alzheimer’s dementia, *PET*  Positron-emission tomography, *SUVR*  Standardized uptake value ratio, *p-tau*  Phosphorylated tau, *CERAD*  Consortium to Establish a Registry for Alzheimer’s Disease Neuropsychological Battery, *FCSRT*  Free and Cued Selective Reminding Test, *MMSE*  Mini-Mental State Examination, *IQR*  Inter-quartile range

### Clinical assessments

The Clinical Dementia Rating and the Consortium to Establish a Registry for Alzheimer’s Disease Neuropsychological Battery (CERAD) [33] were administered by trained psychologists at the LMU hospital memory clinic. A CERAD total score was computed following methods previously described by Chandler et al. (2005) [34], integrating results from six cognitive subtests: semantic fluency (number of animals named in 60 s), modified Boston Naming Test, Word List Learning, Constructional Praxis, Word List Recall, and Word List Recognition Discriminability. Additionally, participants completed the FCSRT, a memory test specifically designed to assess verbal episodic memory [35], as well as the MMSE, which evaluates orientation, memory, attention, and language [36, 37]. The free recall component of the FCSRT is especially sensitive in detecting memory impairments associated with MCI and AD [20]. Together, higher scores on the CERAD battery, FCSRT free recall, and MMSE collectively indicate better cognitive functioning, providing a comprehensive picture of cognitive status alongside neuropathological assessments.

### Cerebrospinal fluid analyses

CSF peptide measures were obtained from aliquoted samples using commercially available enzyme-linked immunosorbent assays (ELISAs; Fujirebio, Malvern, PA). Aβ positivity was defined as a CSF Aβ42/40 ratio of less than 5.5%, following previously established criteria [38]. The concentrations of total tau and *p*-tau181 were measured using Innotest htau-Ag and Innotest *p*-tau ELISA assays (Fujirebio, Europe).

### EEG recordings and stimulus paradigm

**Fig. 1:**
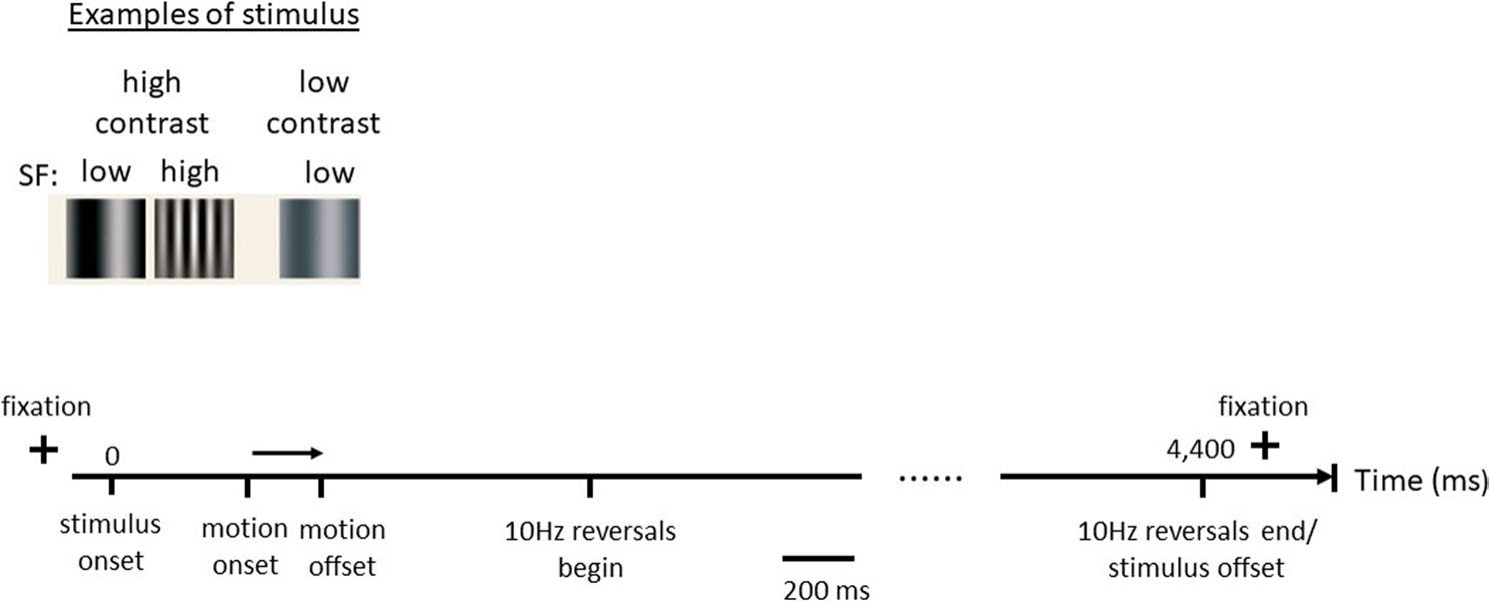
Schematic diagram of the visual stimulation paradigm. Stimuli varied in spatial frequency (SF) and contrast, with 10 Hz counterphase reversals to generate steady-state visual evoked potentials. This paradigm was adapted from the studies by Martínez et al. (2018) and Javitt et al. (2023)

Neural signals were recorded using a 64-channel Waveguard EEG headset (Ag/AgCl electrodes) arranged equidistantly and connected to an ANT recording system (ANT Neuro, Enschede), sampled at 2048 Hz. Participants completed a visual stimulation paradigm [23, 24] comprising a series of distinct visual stimulus types designed to concurrently evaluate various aspects of early visual processing (Fig. 1). This protocol was adapted from prior studies by Martínez et al. (2018) and Javitt et al. (2023). Visual stimuli were displayed against an isochromatic grey background (RGB: 127, 127, 127). Participants were instructed to maintain fixation on a central cross and to ignore the visual stimulus as it was presented. To maintain participant attention, participants were instructed to press the space bar when the fixation cross dimmed slightly (every 3–12 s).

### Automated EEG preprocessing

Collected EEG data were processed using an automated preprocessing pipeline developed in Python, primarily utilizing the MNE library for EEG data analysis. The preprocessing pipeline involved several steps: (1) applying a notch filter (Comb IIR) at 50 Hz and a band-pass filter (Butterworth IIR, 4th order) between 1 and 40 Hz; (2) resampling the EEG data to 256 Hz; (3) marking segments longer than 5 seconds without task-relevant markers as break (bad) segments; (4) identifying bad channels using the RANSAC method implemented in the PyPREP library and interpolating these channels using values from neighboring good channels; (5) detecting and correcting ocular artifacts, including blinks and eye movements, via Independent Component Analysis (ICA), treating the ‘0Z’ channel (a dedicated periorbital electrode provided in the 64-channel Waveguard EEG headset) as an electrooculogram reference for identifying ocular components; (6) identifying and correcting transient, large-amplitude (> 200 µV) artifacts through Principal Component Analysis (PCA); (7) re-referencing EEG signals to the average of all channels; and (8) segmenting the continuous EEG data into epochs from − 0.5 to 4.4 s relative to stimulus onset, applying baseline correction using the − 0.5 to 0 s interval, and discarding epochs exceeding 200 µV peak-to-peak amplitude.

In this study, the upper cutoff of 40 Hz was selected to preserve low-gamma activity relevant to entrainment and spectral features analyzed, while still excluding high-frequency noise. Re-referencing was performed after artifact correction and channel interpolation in accordance with the preprocessing protocol proposed by Ouyang and Li (2025) [39], to reduce the propagation of artifacts during average referencing. On average, 4.7 ± 3 channels per participant were interpolated following automated bad-channel detection. Epochs exceeding 200 µV peak-to-peak amplitude were discarded. A ± 200 µV threshold was used to accommodate both ERP and frequency-domain analyses, in conjunction with ICA- and PCA-based artifact correction. After preprocessing and artifact rejection, at least 50 epochs were retained for all participants and the first 50 of each stimulus condition per participant were used for model training to ensure consistency. Epochs corresponding to each stimulus condition were averaged for each EEG channel. To derive regional EEG measures, epochs were further averaged across groups of spatially adjacent channels. Finally, time-domain EEG metrics—such as event-related potentials, entrainment ratios, spectral connectivity, and entropy—were extracted from various brain regions and analyzed separately according to the specific visual stimuli that elicited them.

### Feature extraction

Specific analyses were conducted on the preprocessed epoch data. For spectral connectivity, magnitude-squared coherence was calculated using MNE’s *spectral_connectivity_epochs* function (method = ‘coh’, mode = ‘multitaper’) and averaged across the 2–40 Hz frequency range (faverage = True). Spectral entropy was evaluated using Shannon entropy to quantify the complexity of the frequency distribution. The entrainment ratio was determined by calculating the power at 10 Hz relative to adjacent frequencies from 1.4 to 3.4 s after trial onset.

ERP analyses included computing peak-centered area-under-the-curve (AUC) features for the P50, N100, N200, and N300 components. ERPs were obtained by averaging across trials and then across channels within each predefined region. For each component, a pre-specified latency window was used (P50: 25–50 ms, N100: 50–150 ms; N200: 150–250 ms; N300: 250–400 ms), within which the most prominent positive or negative peak was identified. The AUC was computed by integrating the ERP waveform within a ± 4-sample window centered on the detected peak (8 samples total; approximately 31 ms at 256 Hz) using Simpson’s rule, yielding a localized measure sensitive to component-specific peak morphology rather than mean amplitude across a broad window. In addition, power of the time–frequency representation (TFR) was computed within fixed latency windows centered on canonical ERP peaks: 100 ms (50–150 ms), 200 ms (150–250 ms), and 300 ms (250–400 ms). Spectral connectivity was analyzed across eight selected channel pairs spanning frontal, central, parietal, and occipital regions. Spectral entropy, entrainment ratio, and ERP features were assessed across channels within 18 predefined sub-regions, as detailed in Appendix Table A.1. The distribution of all the EEG features for ERP-, connectivity, and entropy-derived categories is summarized in Appendix Table A.3.

### Feature selection and regression modelling

**Fig. 2:**
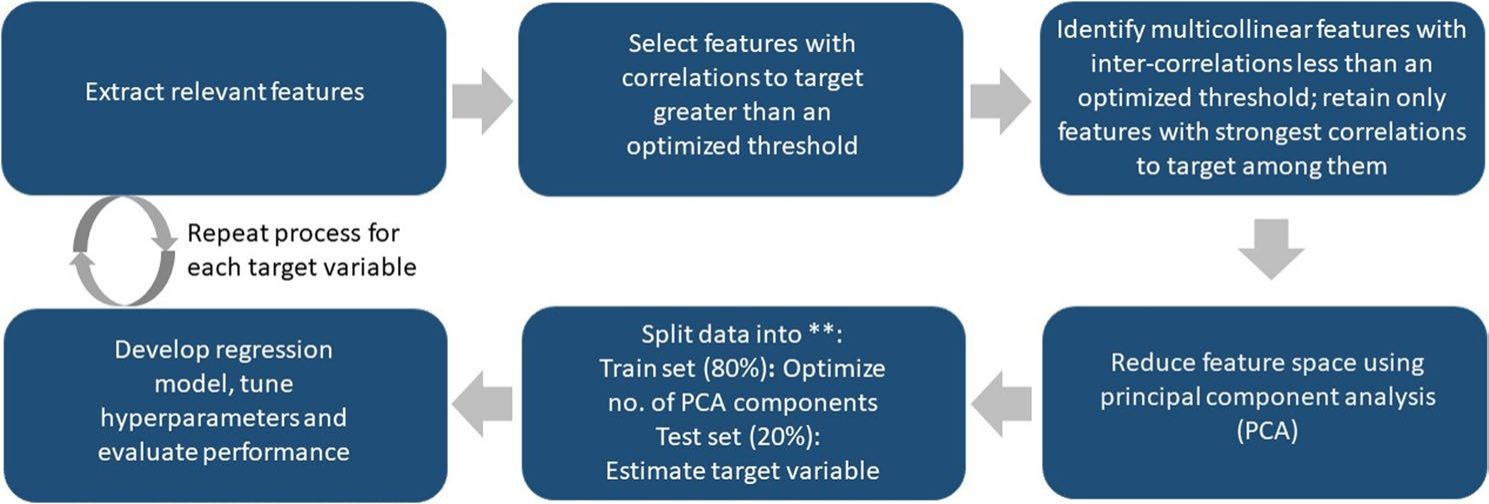
Feature selection and regression modeling pipeline. ^**^The train-test split was balanced and stratified according to participant diagnosis types and PET-amyloid SUVR. The model training and testing process was repeated across five different train-test splits to assess performance consistency

We developed a feature selection and multivariate linear regression pipeline (Fig. 2) in Python, utilizing the MNE, SciPy, and scikit-learn libraries, to estimate PET-amyloid SUVR from neurophysiological EEG signals. Initially, extracted EEG features were evaluated, and those demonstrating correlations with the target variable (i.e., PET-amyloid SUVR, CSF *p*-tau181, FCSRT free recall score, or MMSE score) exceeding an optimized threshold (0.23–0.42, selected for best predictive performance) were retained.

To minimize redundancy and multicollinearity, inter-correlations among the selected features were analyzed, and features were grouped if their inter-correlations surpassed a second optimized threshold (0.6–0.9). From each group, only the feature exhibiting the strongest correlation with the target variable was retained. These threshold ranges were explored systematically to assess robustness across parameter choices rather than to optimize a single configuration.

Participants were pseudo-randomly divided into training (80%) and test (20%) subsets, stratified based on diagnosis and PET-amyloid SUVR (for estimation of PET-amyloid SUVR, FCSRT free recall, and MMSE scores) or CSF *p*-tau181 levels (for estimation of CSF *p*-tau181) to ensure similar distributions across subsets. Within each split, all preprocessing steps, including feature scaling and PCA, were learned exclusively from the training data and subsequently applied to the corresponding test data.

Dimensionality reduction was performed using PCA on the training data, retaining 8–16 principal components explaining approximately 60–70% of the variance. A Ridge regression model was then trained on the PCA-transformed training data, with the regularization parameter optimized via cross-validation confined to the training set. For each split and parameter configuration, predictive performance metrics were computed separately for the training and test sets and recorded for comparison.

### Power analysis

Given the modest sample size and the predictive modeling framework employed, we performed a sensitivity power analysis using Fisher’s *z* transformation for the reported Spearman’s correlation between predicted and true values. Using Fisher’s *z* transformation (two-sided α = 0.05), the available sample sizes of 38–48 (depending on the target variable) provides 80% power to detect correlations of approximately *r* ≥ 0.43–0.48. This analysis was used to contextualize the reliability of the observed correlations rather than to determine sample size a priori.

## Results

### ERP amplitudes elicited by visual stimuli varied as a function of the diagnostic group

**Fig. 3:**
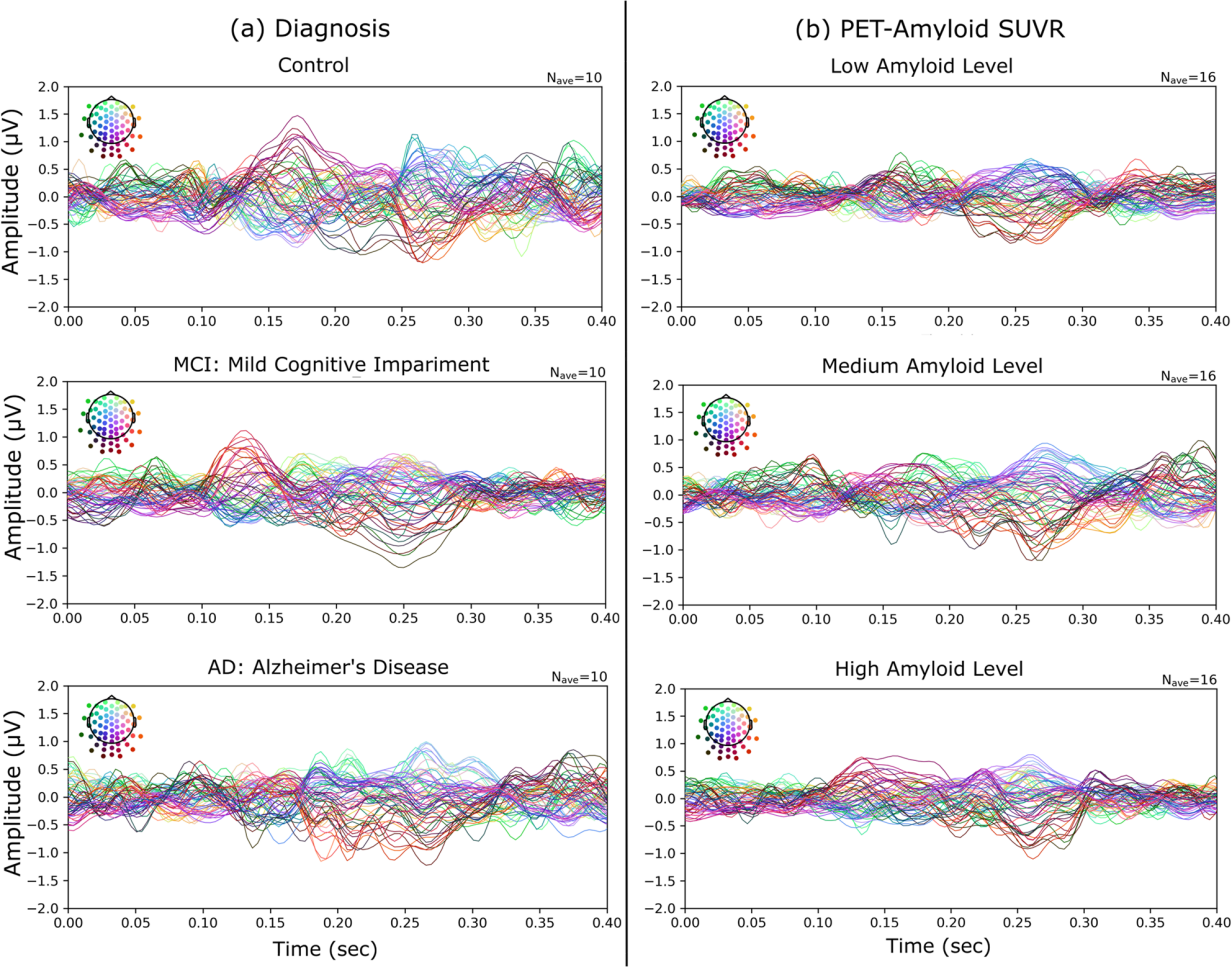
Channel-wise grand-average ERPs for one visual stimulus, stratified by (**a**) diagnostic group (controls, MCI, AD; *n* = 10 per group) and (**b**) PET-amyloid SUVR level (low, medium, high; *n* = 16 per level). Each trace represents the ERP waveform for a single EEG channel, obtained by averaging across trials and participants within the corresponding group. Channels are color-coded according to scalp location (inset). This visualization illustrates the spatial distribution and inter-channel variability of evoked responses; quantitative group comparisons are based on extracted ERP and time–frequency features

ERPs corresponding to the same stimulus condition were averaged across subsets of participants stratified by diagnostic group (control, MCI, and AD) or PET-amyloid SUVR level (high, mid, and low) to obtain grand average ERPs for each EEG channel. For instance, the grand average ERPs associated with one of the stimulus conditions were compared across different diagnostic groups (e.g., grand averages of 10 participants in each group shown in Fig. 3a) and PET-amyloid SUVR levels (e.g., grand averages of 16 participants in each level shown in Fig. 3b). Differences in ERP amplitude served as direct indicators of the functional integrity of neurons targeted by the respective stimulus. A trend was observed, showing reduced ERP amplitudes in participants with AD compared to controls, which was evident during the 0 to 0.4-sec time interval in Fig. 3a.

### Regression modeling of selected ERP features estimated PET-amyloid SUVR with higher precision than CSF amyloid ratio

Five different train-test set splits were generated by stratifying based on the diagnostic group and PET-amyloid SUVR. Table 2 presents the training and test results obtained by repeating the regression modeling procedures on these 5 train-test splits. Correlations higher than 0.79 were observed in all train-test splits, indicating stable associations under varying data partitions.

**Table 2:** Correlation coefficient (*Corr*), *p*-values (*p*), and mean absolute percentage error (*MAPE*) across 5 different train-test splits for PET-amyloid SUVR

Train- test	All (combined) Corr *p* MAPE			Train Corr *p* MAPE			Test Corr *p* MAPE		
Set 1	0.865	< 0.01	0.110	0.871	< 0.01	0.100	0.834	< 0.01	0.143
Set 2	0.892	< 0.01	0.099	0.889	< 0.01	0.100	0.794	< 0.01	0.095
Set 3	0.863	< 0.01	0.103	0.876	< 0.01	0.099	0.794	< 0.01	0.119
Set 4	0.857	< 0.01	0.107	0.849	< 0.01	0.103	0.900	< 0.01	0.120
Set 5	0.874	< 0.01	0.117	0.885	< 0.01	0.109	0.818	< 0.01	0.144

In set 2, which is shown as a representative example, a list of 243 features was identified using the feature selection method depicted in Fig. 2. PCA optimally reduced these selected features to 12 principal components (PCs), which collectively explained approximately 65% of the variance. The multivariate linear regression model was trained using these PCs and the true PET-amyloid SUVR from participants in the training set, and the learned model parameters were applied to the corresponding test set to generate PET-amyloid SUVR estimates. Because feature selection was performed using the full dataset, the reported correlations reflected internally validated associations rather than fully independent out-of-sample performance. Accordingly, train–test splits were used to assess stability across data partitions rather than strict generalizability.

As a control analysis, PET-amyloid SUVR values in the training sets were randomly shuffled (50 repetitions per split) while preserving the original data structure. Under this permutation procedure, the resulting correlation coefficients for the combined training and test sets were consistently below 0.5 across all splits (Appendix Table A.2), indicating that the observed performance in the unshuffled data is unlikely to arise by chance.

**Fig. 4:**
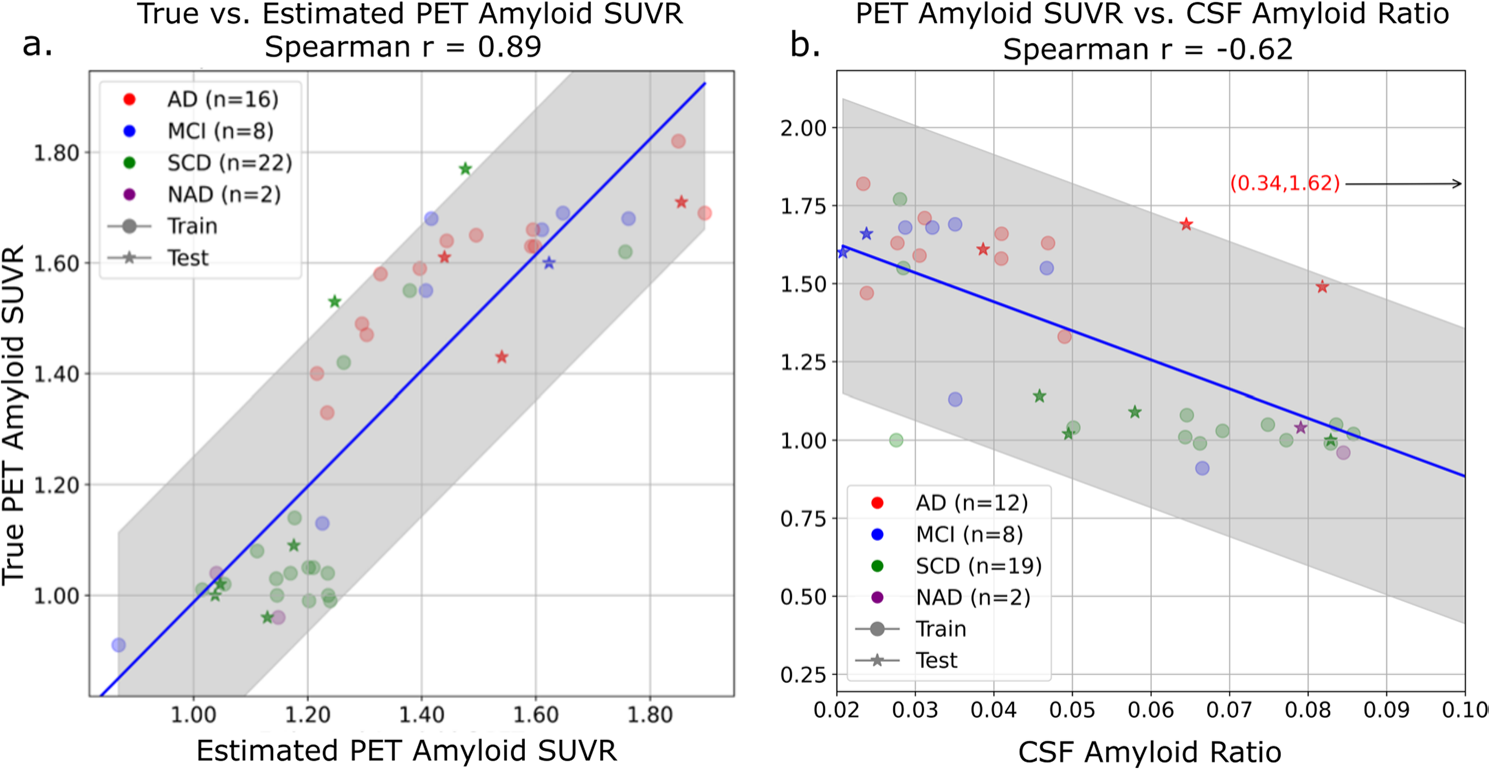
The regression model trained with the principal components generated from the selected ERP features performed better than the CSF amyloid ratio alone in estimating PET-amyloid SUVR. **a** Spearman correlation (*r* = 0.89, *p* < 0.01) between true and estimated PET-amyloid SUVR from the regression model developed using the train-test set 2 (Table 2). **b** Spearman correlation (*r* = -0.62, *p* < 0.05) between PET-amyloid SUVR and CSF amyloid ratio across all participants. Participants are labeled by diagnostic types using different colors and by train and test sets using different marker types. One extreme value falls outside the displayed axis limits and is therefore not shown for visualization purposes; this data point was included in all analyses and model evaluations. The gray area indicates the 95% prediction interval

The Spearman correlation in the combined training and test sets for train-test set 2 was found to be *r* = 0.89 with a p-value < 0.01 between true and estimated PET-amyloid SUVR (Fig. 4a). For comparison, in Fig. 4b, the Spearman correlation between PET-amyloid SUVR and CSF amyloid ratio for all participants was − 0.62 (*p* < 0.05). The absolute value of this correlation coefficient is lower than the correlation coefficient derived from the regression modeling. Hence, the PCs of the selected ERP features used in the regression model were better at estimating PET-amyloid SUVR than using the CSF amyloid ratio alone as the predictor.

While scatter plots in Fig. 4 display predictions from both training and test sets for visualization of the overall relationship between estimated and true values, quantitative performance metrics were computed separately for the training and test subsets within each split and are reported in Table 2. Consistency of these metrics across splits further supports the stability of the observed associations. The same evaluation strategy was applied to the estimation of the other biomarkers presented below.

**Fig. 5:**
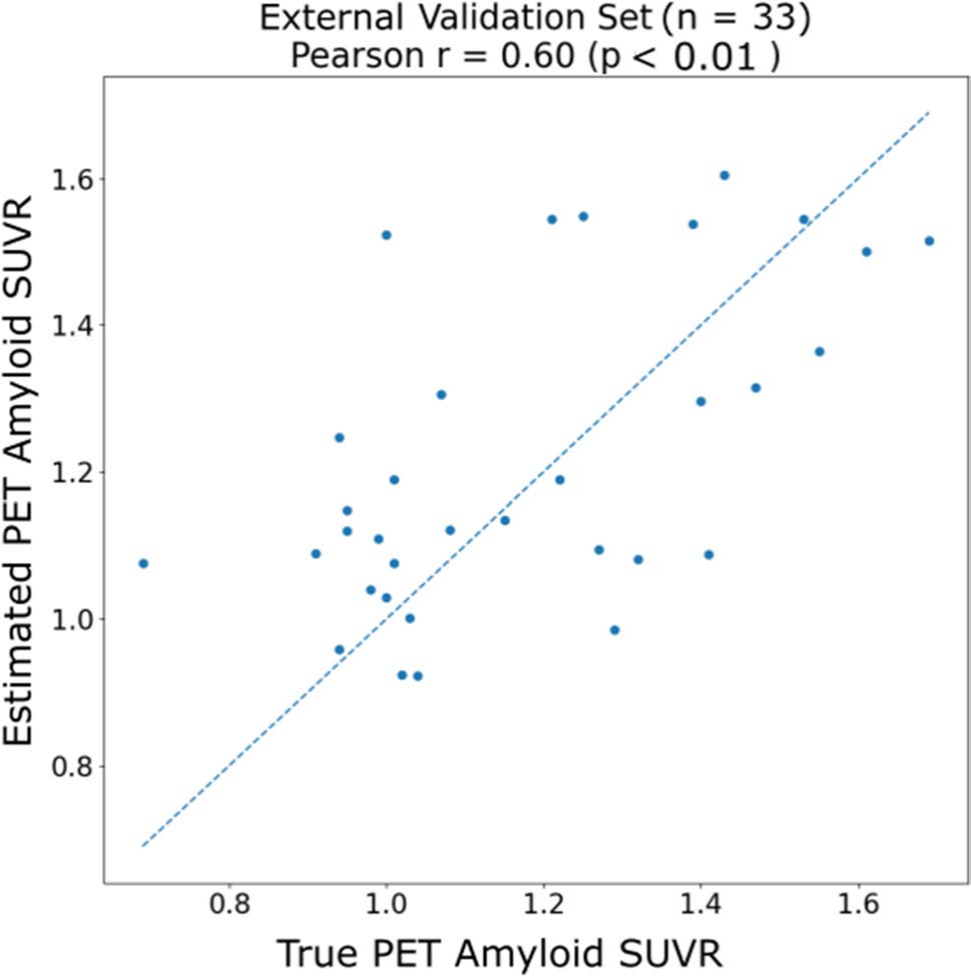
External validation of PET-amyloid SUVR estimation using EPT-derived features. Scatter plot showing the relationship between true and estimated PET-amyloid SUVR values in an independent validation cohort (*n* = 33). The dashed line represents the identity line (y = x). Estimations were generated using a model refit on features selected from the five splits for PET-amyloid SUVR estimation in the primary dataset, using all available samples from that dataset. A moderate and statistically significant correlation was observed (Pearson’s *r* = 0.60, *p* < 0.01), with a mean absolute percentage error of 0.15, indicating preserved predictive signal in an external cohort

As an exploratory sensitivity analysis, we also examined the association between predicted and true PET-amyloid SUVR after adjusting for global cognitive performance, i.e., MMSE. This analysis yielded a similar association between predicted and true PET-amyloid SUVR in test subjects from train–test split 2 (Appendix Table A.3). Moreover, to further assess generalizability, we performed an exploratory validation using an independent external cohort (*n* = 33). To ensure consistency with the primary analysis, features selected from the five splits for PET-amyloid SUVR estimation were used to refit a regression model using all available LMU samples, and the model was then applied to the external cohort, which was independently acquired outside the LMU dataset. In this external validation set, estimated PET-amyloid SUVR values remained significantly associated with true SUVR values (Pearson’s *r* = 0.60, *p* < 0.01; Fig. 5), although attenuated compared to the internally validated results.

### Strong correlations between true and estimated CSF p-tau181 levels using regression modeling of selected ERP features

**Fig. 6:**
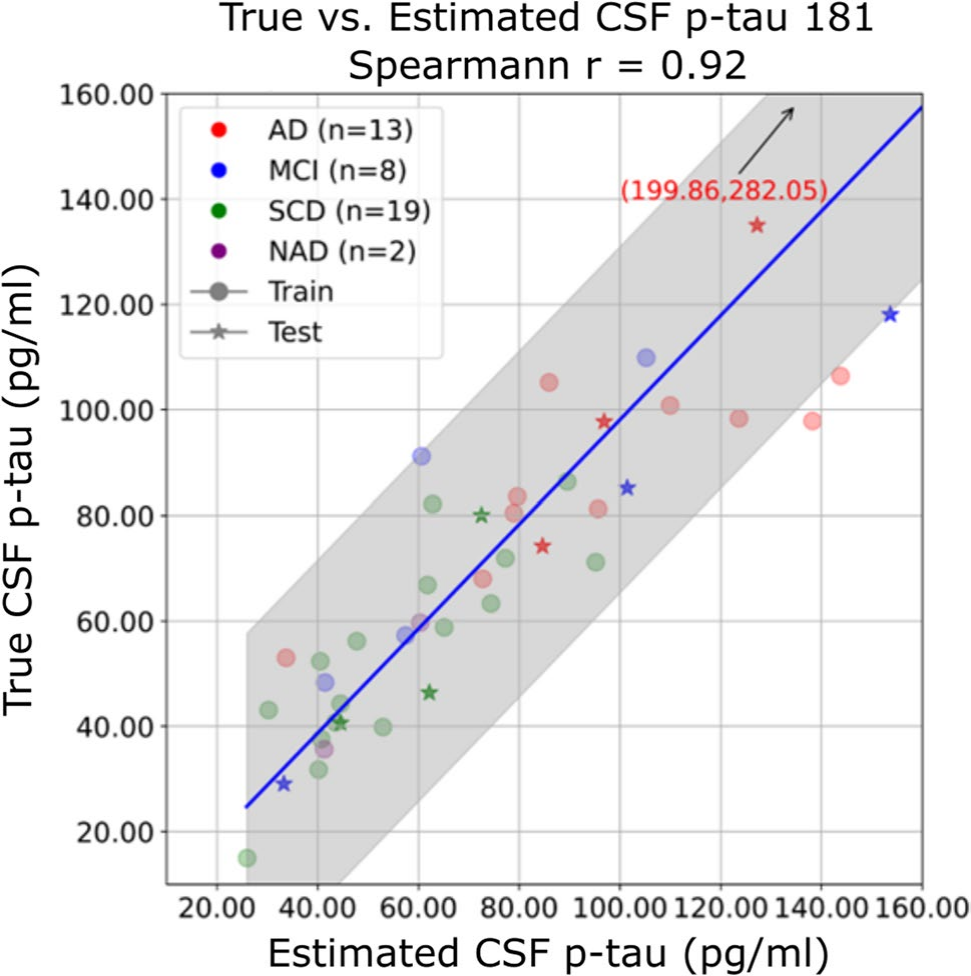
Estimation of CSF *p*-tau181. Spearman correlation (*r* = 0.92, *p* < 0.01) between true and estimated CSF *p*-tau181 levels from the regression model developed using the train-test set 3 (Table 3). Participants are labeled by diagnostic types using different colors and by train and test sets using different marker types. One extreme value falls outside the displayed axis limits and is therefore not shown for visualization purposes; this data point was included in all analyses and model evaluations. The gray area indicates the 95% prediction interval

We followed similar procedures, as illustrated in Fig. 2, to train and evaluate regression models to estimate CST *p*-tau181 levels. Participants were divided into training and testing datasets through five separate splits, stratified according to diagnostic groups and CSF *p*-tau181 levels. Table 3 summarizes the results from these regression analyses across all five train-test splits, with correlation coefficients ranging from 0.86 to 0.92. The best performance was achieved in set 3, yielding the highest correlation. In this train-test set, 202 relevant features were identified through the feature selection process. These features were subsequently reduced using PCA to 10 PCs, explaining approximately 64% of the total variance. A multivariate linear regression model was trained on these PCs using the participants’ true CSF *p*-tau181 values from the training set. The trained model was then validated on the test dataset using the same PCs to estimate CSF *p*-tau181 levels. Figure 6 depicts the Spearman correlation (*r* = 0.92, *p* < 0.01) for the combined train and test sets, demonstrating strong predictive performance.

**Table 3:** Correlation coefficient (*Corr*), *p*-values (*p*), and mean absolute percentage error (*MAPE*) across 5 different train-test splits for CSF *p*-tau181

Train- test	All (combined) Corr *p* MAPE			Train Corr *p* MAPE			Test Corr *p* MAPE		
Set 1	0.891	< 0.01	0.238	0.908	< 0.01	0.240	0.817	< 0.01	0.232
Set 2	0.890	< 0.01	0.275	0.894	< 0.01	0.273	0.883	< 0.01	0.286
Set 3	0.918	< 0.01	0.179	0.909	< 0.01	0.186	0.950	< 0.01	0.152
Set 4	0.877	< 0.01	0.256	0.872	< 0.01	0.261	0.850	< 0.01	0.234
Set 5	0.869	< 0.01	0.224	0.867	< 0.01	0.198	0.817	< 0.01	0.319

### Estimation of cognitive performance (FCSRT free recall and MMSE scores) via regression modeling of ERP features

**Fig. 7:**
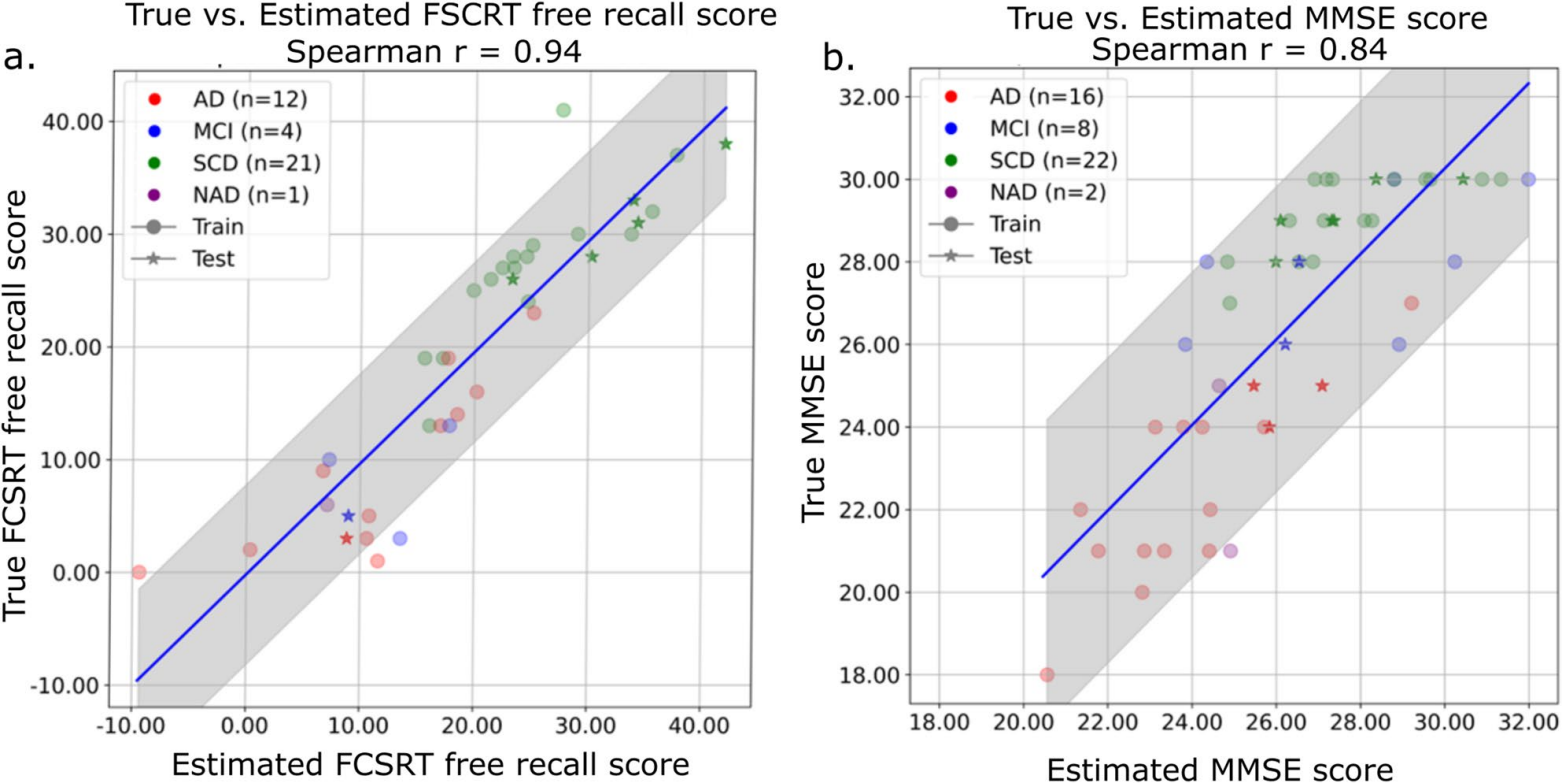
Estimation of FCSRT free recall and MMSE scores. (a) Spearman correlation (*r* = 0.94, *p* < 0.01) between true and estimated FCSRT free recall scores from the regression model developed using the train-test set 1 (Table 4). (b) Spearman correlation (*r* = 0.83, *p* < 0.01) between true and estimated MMSE scores from the regression model developed using the train-test set 1 (Table 5). Participants are labeled by diagnostic types using different colors and by train and test sets using different marker types. The gray area indicates the 95% prediction interval

We applied similar procedures to train and evaluate multivariate linear regression models for estimating the FCSRT free recall and MMSE scores. Utilizing the same five train-test splits as used for PET-amyloid SUVR estimations, the results for the regression models are summarized in Table 4 (FCSRT free recall) and Table 5 (MMSE). For the FCSRT free recall scores, the highest correlation coefficient was achieved in set 1. In this split, feature selection identified 338 relevant features, which were subsequently reduced into 11 PCs using PCA, collectively explaining approximately 66% of the variance. Similar to PET-amyloid SUVR estimations, a multivariate linear regression model was developed using these PCs along with the true FCSRT free recall scores from the training participants and was subsequently validated on the test participants using the same set of PCs to estimate their FCSRT free recall scores. Figure 7a illustrates the Spearman correlation (*r* = 0.94, *p* < 0.01) for the combined training and testing datasets, demonstrating robust predictive performance.

**Table 4:** Correlation coefficient (*Corr*), *p*-values (*p*), and mean squared error (*MSE*) across 5 different train-test splits for FCSRT free recall

Train- test	All (combined) Corr *p* MAPE			Train Corr *p* MAPE			Test Corr *p* MAPE		
Set 1	0.941	< 0.01	24.75	0.937	< 0.01	27.53	0.964	< 0.01	12.40
Set 2	0.870	< 0.01	42.37	0.859	< 0.01	39.60	0.870	< 0.01	51.31
Set 3	0.937	< 0.01	28.90	0.935	< 0.01	31.32	0.898	< 0.01	19.82
Set 4	0.942	< 0.01	26.39	0.950	< 0.01	17.57	0.874	< 0.01	59.47
Set 5	0.943	< 0.01	25.65	0.941	< 0.01	23.36	0.95	< 0.01	33.06

**Table 5:** Correlation coefficient (*Corr*), p-values (*p*), and mean absolute percentage error (*MAPE*) across 5 different train-test splits for MMSE

Train- test	All (combined) Corr *p* MAPE			Train Corr *p* MAPE			Test Corr *p* MAPE		
Set 1	0.841	< 0.01	0.066	0.848	< 0.01	0.058	0.752	< 0.01	0.091
Set 2	0.828	< 0.01	0.070	0.824	< 0.01	0.066	0.938	< 0.01	0.085
Set 3	0.828	< 0.01	0.070	0.793	< 0.01	0.071	0.938	< 0.01	0.064
Set 4	0.830	< 0.01	0.068	0.825	< 0.01	0.072	0.776	< 0.01	0.054
Set 5	0.822	< 0.01	0.069	0.807	< 0.01	0.063	0.734	0.01	0.086

Meanwhile, for the MMSE scores, the highest correlation coefficient was again observed in set 1. In this set, the feature selection procedure yielded 170 relevant features, which were then reduced to 9 PCs via PCA, explaining approximately 60% of the total variance. A multivariate linear regression model trained with these PCs and true MMSE scores from the training participants was evaluated on the test set. Figure 7b demonstrates the Spearman correlation (*r* = 0.84, *p* < 0.01) of the combined train and test sets.

## Discussion

Healthcare providers face significant challenges in effectively treating dementia, largely because common initial symptoms often mask a spectrum of diverse, progressive diseases that are difficult to differentiate, monitor, and specifically address. To tackle these difficulties, the Amyloid, Tau, Neurodegeneration (ATN) diagnostic framework was established to enhance AD detection and monitoring [40]. Nevertheless, the practical implementation of the ATN framework and similar diagnostic models remains challenging due to the absence of a single portable technology capable of simultaneously assessing all necessary biomarkers.

One promising early indicator of AD pathology is altered neural activity. In humans, combined MRI and PET imaging studies have demonstrated correlations among cortical Aβ accumulation, early synaptic loss, and changes in global functional connectivity occurring before cognitive symptom onset [12]. Resting-state EEG analyses have also identified similar cortical network alterations linked to AD. However, despite these insights, resting-state EEG measures currently lack the sensitivity and specificity required for widespread adoption as screening or clinical decision-support tools.

To address these limitations, the present study utilizes the EPT protocol coupled with an automated pipeline. This protocol facilitates sensitive interneuron evaluation by employing targeted, sequential stimulations across spatially distinct neural pathways [23, 41], thereby generating a functional map of interneuron integrity. Building upon previous research demonstrating EEG’s predictive capability for amyloid positivity using this protocol, we integrated it with an automated EEG preprocessing and feature extraction pipeline to estimate PET-amyloid SUVR and other related biomarkers of AD (i.e., CSF *p*-tau181, FCSRT free recall, and MMSE scores). Our study validated that this integrated approach captures neural features associated with AD-related biomarkers in a clinically enriched cohort. Specifically, the pipeline estimated PET-amyloid SUVR values with strong correlations (*r* > 0.8) between actual and predicted values, surpassing the predictive performance of CSF amyloid ratios alone. Previous research by Wisch et al. (2022) estimated continuous PET-amyloid values from CSF amyloid ratios using linear and generalized additive models, resulting in mean absolute percentage errors (MAPEs) of 23.8% and 15.1%, respectively [42]. In contrast, the best MAPE that we achieved using our regression modeling pipeline was 10%. Additionally, applying the same procedures and pipeline to estimate CSF *p*-tau181 levels, FCSRT free recall scores, and MMSE scores resulted in similarly robust correlations (0.8–0.94) with their true values. These findings demonstrate that the integration of the EPT protocol and our automated pipeline provides a powerful method that enables the estimation of multiple important AD biomarkers.

Because EEG-derived features also predicted cognitive outcomes, we performed an exploratory sensitivity analysis to assess whether the observed association between EEG-based predictions and amyloid burden was driven primarily by global cognitive impairment. In test subjects from train–test split 2, MMSE was moderately correlated with true PET-amyloid SUVR (Spearman’s *r* ≈ − 0.6, *p* = 0.045), close to the expected relationship between cognitive impairment and amyloid burden in a clinically enriched cohort. Importantly, after adjusting for MMSE, the association between predicted and true PET-amyloid SUVR remained strong and statistically significant (Spearman’s *r* = 0.76, *p* = 0.011), with only modest attenuation relative to the unadjusted correlation (*r* = 0.79; Appendix Table A.3). This finding suggests that the EEG–amyloid association is not fully explained by global disease severity, while acknowledging that shared variance between cognition and amyloid burden is expected in this cohort.

From a clinical perspective, the ability to estimate multiple AD-relevant biomarkers from a single, brief EEG session has several potential implications. EPT-based measures may be particularly useful in settings where access to PET imaging or lumbar puncture is limited, serving as a functional screening or triage tool to identify individuals who may benefit from confirmatory testing. In research contexts, EPT could facilitate more efficient cohort enrichment for clinical trials by providing a scalable, non-invasive assessment of functional disease burden. Importantly, because EEG reflects real-time neural dynamics, EPT-derived features may offer complementary insights into disease progression and treatment response that are not captured by static molecular biomarkers alone.

Recent advances in blood-based biomarkers, including Single Molecular Array-based assays for *p*-tau and amyloid species [43, 44], have substantially improved the feasibility of early AD detection and large-scale screening and are increasingly used as scalable rule-out tools in clinical workflows. However, these peripheral measures primarily reflect downstream molecular pathology and do not directly capture functional consequences at the neural circuit level, often necessitating confirmatory PET imaging with associated cost and false-positive burden. Reported correlations between plasma amyloid measures and PET-amyloid SUVR are typically moderate (approximately *r* = − 0.5 to − 0.6) [45, 46], highlighting the limitations of peripheral biomarkers for precise estimation of cerebral amyloid burden. The proposed EEG-based EPT approach is therefore positioned not as a replacement for blood biomarkers, but as a complementary, upstream functional measure that directly probes interneuron dynamics and network-level integrity. In this framework, EPT may serve as a next-step assessment following blood positivity to refine risk stratification and reduce unnecessary and expensive PET imaging, while also offering particular value for longitudinal monitoring and treatment response, where changes in neural dynamics may provide sensitive functional endpoints that precede or diverge from molecular biomarkers.

However, this study has several limitations. The modest sample size (38–48 participants, depending on the target variable) and cross-sectional design limit generalizability and preclude longitudinal analyses and fine-grained subgroup comparisons. In addition, because features from multiple EEG domains were selected jointly and subsequently transformed using PCA, effect sizes for individual features or feature categories are not directly interpretable within the present modeling framework. Sensitivity power analyses indicated approximately 80% power to detect correlations of *r* ≈ 0.43–0.48 given the available sample sizes. Across target variables, the observed associations between true and estimated biomarker values in the combined full training and test sets were generally larger than 0.75, substantially exceeding these thresholds. This suggests that the primary limitation of the present study is not detectability of the reported effects, but rather uncertainty in generalizability and the need for independent validation. Moreover, feature selection was performed using the full dataset, which may lead to optimistic performance estimates. Accordingly, the reported findings should be interpreted as internally validated and preliminary, pending confirmation in larger, fully independent validation cohorts to establish robustness, specificity across etiologies, and clinical utility. To partially address this limitation, we conducted an exploratory validation in an independent external cohort using features selected from the five splits for PET-amyloid SUVR estimation. A regression model was refit using these features and applied to the external cohort. Although predictive performance was reduced compared to the internally validated results, the association between EEG-derived estimations and PET-amyloid SUVR remained statistically significant, suggesting that the observed relationships are not solely driven by overfitting to the original dataset. Notably, the explained variance decreased from approximately 79% (*r* = 0.89²) in the primary dataset to 36% (*r* = 0.60²) in the external cohort, highlighting a performance gap between internal and external validation. This reduction suggests that further refinement and validation in larger, diverse cohorts are required. The attenuation in performance likely reflects differences in cohort characteristics and sample size, as well as potential domain shift between datasets.

Additionally, due to the invasive nature of the clinical assessments required, no healthy controls were explicitly recruited. The participants represented a biased cohort with significant cognitive concerns warranting clinical PET scans, thus having a higher likelihood of elevated amyloid levels sufficient to explain cognitive deficits. This cohort bias restricts the generalizability of the findings and limits conclusions regarding potential benefits for population-wide screening. Moreover, the necessary exclusion criteria (e.g., older adults without complex neurological histories, such as prior strokes) further limit the general applicability of these findings. Consequently, the diagnostic specificity of EPT with respect to alternative neurodegenerative etiologies could not be evaluated in this cohort. Despite these limitations, the results from this clinical study strongly suggest that key diagnostic criteria for AD can be reliably estimated using a single 30-minute EEG session.

AD remains a critical global health challenge, emphasizing the urgent need for reliable, portable, and non-invasive methods for early characterization of AD-related pathology and disease staging. Identifying AD pathophysiology prior to the onset of cognitive symptoms could provide valuable opportunities for initiating preventative interventions. Altered neural activity, observable via techniques such as EEG and ERPs, holds substantial promise as a non-invasive biomarker for AD. Specifically, EPT demonstrated an effective correlation between neural features and multiple AD biomarkers. Given EEG’s portability and non-invasive nature, this EPT protocol may support scalable estimation of AD-related biomarkers, particularly as a complementary tool within clinically enriched settings. Further research and validation of this protocol as a screening methodology could significantly enhance clinical care and research in AD, enabling earlier detection, more precise cohort selection, and targeted therapeutic interventions. In addition, future studies incorporating larger cohorts with diverse neurodegenerative and neurological conditions will be essential to determine the diagnostic specificity of EPT and its role in differential diagnosis.

## Supplementary Information

Supplementary Material 1.

## Data Availability

All data produced in the present study are available upon reasonable request to the authors.

## Abbreviations

AD: Alzheimer’s disease
ATN: Amyloid, Tau, Neurodegeneration
AUC: Area under the curve
Aβ: Amyloid-β
CERAD: Consortium to Establish a Registry for Alzheimer’s Disease Neuropsychological Battery
CSF: Cerebrospinal fluid
EEG: Electroencephalography
ELISAs: Enzyme-linked immunosorbent assays
EPT: Evoked Potential Tomography
ERP: Event-related potential
FCSRT: Free and Cue Selecting Reminding Test
LMU: Ludwig-Maximilians-University
MCI: Mild cognitive impairment
MMSE: Mini-Mental State Examination
MRI: Magnetic resonance imaging
NAD: Non-Alzheimer’s dementia
PCA: Principal component analysis
PET: Positron-emission-tomography
p-tau: Phosphorylated tau
SCD: Subjective cognitive decline
SUVR: Standardized uptake value ratio
TFR: Time-frequency representation
