# Supplementary material for: Estimation of positron emission tomography amyloid load and related biomarkers in Alzheimer’s disease using evoked potential tomography EEG: development and internal validation in a cross-sectional cohort
Source: Alzheimers Res Ther. 2026 Jun 12;18:147. doi: 10.1186/s13195-026-02076-7 (PMC13267758; doi:10.1186/s13195-026-02076-7)
Supplement: Supplementary file 1 — Supplementary Material 1. [file 13195_2026_2076_MOESM1_ESM.docx]

**Appendix**

**Table A.1: Eighteen defined sub-regions and their corresponding channel names in event response potential analysis.**

| **Sub-regions** | **Name of Channels** |
| --- | --- |
| Frontal | 1Z, 2Z, 3Z, 2L, 2R, 3L, 3R |
| Central | 3Z, 4Z, 5Z, 4L, 4R, 5L, 5R |
| Parietal | 5Z, 6Z, 7Z, 6L, 6R, 7L, 7R |
| Occipital | 7Z, 8Z, 9Z, 8L, 8R, 9L, 9R, 10L, 10R |
| Right Frontal | 1RA, 1RB, 1RC, 2RB |
| Left Frontal | 1LA, 1LB, 1LC, 2LB |
| Right Central | 4R, 5R |
| Left Central | 4L, 5L |
| Right Parietal | 6R, 7R |
| Left Parietal | 6L, 7L |
| Right Occipital | 8R, 9R, 10R |
| Left Occipital | 8L, 9L, 10L |
| Right Anterior Temporal | 1RD, 2RC, 2RD |
| Left Anterior Temporal | 1LD, 2LC, 2LD |
| Right Medial Temporal | 2RA, 3RA, 3RB, 4RB, 3RC |
| Left Medial Temporal | 2LA, 3LA, 3LB, 4LB, 3LC |
| Right Parietal Temporal | 5RB, 4RC, 5RC, 3RD, 4RD |
| Left Parietal Temporal | 5LB, 4LC, 5LC, 3LD, 4LD |

**Table A. 2. Correlation coefficients (Corr), *p*-values (*p*), and mean absolute percentage errors (MAPE) for 5 train-test splits using shuffled PET-amyloid SUVR values in the training sets.**

| **Train-**  **test** | **All (combined)**  ***Corr           p           MAPE*** | | | **Train**  ***Corr            p         MAPE*** | | | **Test**  ***Corr          p             MAPE*** | | |  |
| --- | --- | --- | --- | --- | --- | --- | --- | --- | --- | --- |
| **Set 1** | 0.453 | < 0.01 | 0.176 | 0.553 | < 0.01 | 0.168 | -0.050 | 0.342 | 0.205 | |
| **Set 2** | 0.426 | 0.016 | 0.178 | 0.504 | < 0.01 | 0.167 | 0.061 | 0.270 | 0.221 | |
| **Set 3** | 0.450 | 0.019 | 0.176 | 0.556 | < 0.01 | 0.162 | -0.016 | 0.349 | 0.223 | |
| **Set 4** | 0.434 | 0.013 | 0.175 | 0.531 | < 0.01 | 0.164 | -0.057 | 0.459 | 0.214 | |
| **Set 5** | 0.450 | 0.018 | 0.179 | 0.540 | < 0.01 | 0.167 | 0.046 | 0.296 | 0.217 | |

**Table A. 3. Distribution of all EEG features by domain (ERP, spectral connectivity, and entropy).**

| **Feature domain** | **Number of features** | **Percentage of total** |
| --- | --- | --- |
| ERP-derived | 13257 | 97.57 % |
| Spectral connectivity-derived | 240 | 1.77 % |
| Entropy-derived | 90 | 0.66 % |
| **Total** | **13587** | **100 %** |

**Table A. 4. Exploratory sensitivity analysis controlling for cognition, i.e., MMSE (test set of split 2).** Partial correlations were computed by regressing estimated and true PET-amyloid SUVR values separately on MMSE and correlating the resulting residuals using Spearman’s rank correlation. The MMSE and true PET-amyloid SUVR association is shown for contextual reference. Analyses are exploratory.

| **Analysis** | **Spearman’s r** | **p-value** |
| --- | --- | --- |
| Estimated vs. true SUVR (unadjusted) | 0.79 | < 0.01 |
| MMSE vs. true SUVR | -0.64 | 0.045 |
| Partial correlation (MMSE-adjusted) | 0.76 | 0.011 |
